# Treatment of Moderate to Severe Psoriasis during the COVID-19 Pandemic: Lessons Learned and Opportunities

**DOI:** 10.3390/jcm11092422

**Published:** 2022-04-26

**Authors:** Anna Campanati, Federico Diotallevi, Emanuela Martina, Giulia Radi, Annamaria Offidani

**Affiliations:** Dermatologic Clinic, Department of Clinical and Molecular Sciences, Polytechnic Marche University, 60200 Ancona, Italy; federico.diotallevi@gmail.com (F.D.); radigiu1@gmail.com (G.R.); a.offidani@ospedaliriuniti.marche.it (A.O.)

**Keywords:** psoriasis, COVID-19, SARS-CoV-2, biologics, vaccines, systemic treatments

## Abstract

Since the beginning of the coronavirus disease 2019 (COVID-19) pandemic, clinicians have been overwhelmed by questions beyond the SARS-CoV-2 infection itself. In dermatology practice, clinicians have been facing difficulties concerning therapeutic management of chronic immune-mediated skin disease, above all psoriasis. Major challenges arisen were to understand the role of immunosuppression or immunomodulation on COVID-19 evolution, the benefit/risk ratio related to discontinuation or modification of ongoing treatment, and the appropriateness of initiating new treatments, the optimization of timing in vaccination administration to patients under immunomodulatory treatments, and finally how to find new strategy of patients’ management through remote assistance. In this comprehensive review, we present the current evidence about the course and management of psoriasis during the COVID-19 pandemic. The general message from dermatologists was that data did not suggest that having PSO or its treatment significantly increased risk of SARS-CoV-2 infection or more severe COVID-19 course, the vaccination is highly recommended in all psoriatic patients, beyond ongoing treatment, and that the telehealth experience was a success overall.

## 1. Introduction

Moderate to severe psoriasis is a chronic systemic inflammatory disease whose prevalence in Europe is about 2–3% of general population.

The coronavirus disease 2019 (COVID-19) pandemic has led clinicians to explore the effect of SARS-CoV-2 infection on psoriasis course and management. 

Starting from February 2020, with the pandemic progressively spreading throughout Europe, our understanding of the impact of COVID-19 on psoriasis (and of psoriasis treatment on COVID-19) is rapidly evolving. 

There are many reasons why COVID can have crucial effects on psoriasis: psoriasis is a chronic disease whose clinical course can be exacerbated by a viral infection. Psoriasis is associated with the metabolic syndrome which can be a risk factor for evolution to a severe form of COVID-19. Patients with severe forms are frequently on immunosuppressive treatment, with potential negative repercussions on the evolution of COVID-19. 

For all these reasons, psoriatic patients could be considered as frail patients [1] therefore they should be vaccinated with priority over other categories of subjects; however the worsening role of vaccinations on the progress of psoriasis is widely reported in the literature, thus vaccine hesitation is common among psoriatic patients. In this scenario, several concerns have been raised regarding management of patients with psoriasis during pandemic: which can be the impact of SARS-CoV-2 infection on psoriasis clinical course? Does psoriasis influence the risk to evolve toward serious COVID-19 in SARS-CoV-2-infected patients? How SARS-CoV-2 infection can change our management of psoriasis patients? What dermatologists should know on SARS-CoV-2 vaccination for psoriasis patients, and how they can fight vaccinal hesitation?

All these topics will be considered in the light of the most recent literature data, to provide useful information on a practical level to all dermatologists who manage psoriasis patients daily in this pandemic healthcare context.

## 2. Materials and Methods

This systematic review was based on the approach developed by Arksey and O’Malley [2,3] that includes five essential steps: identification of the research question; identification of appropriate studies; selection of studies; tracking of data; and collection, summarization, and reporting of results. The Preferred Reporting Items for Systematic Reviews and Meta-Analysis (PRISMA) extension for scoping review criteria was used to guide the conduction and reporting of the review.

### 2.1. Identification of the Research Question

A brainstorming approach involving the entire research team was used to identify the research questions. The research group included five dermatologists with expertise in psoriasis.

At the initial meeting, the research group identified the research question and determined the research strategy. The research question was: “how COVID-19 pandemic can influence clinical expression and management of psoriasis?”

### 2.2. Study Selection Process

We performed a worldwide systematic review of studies reporting on COVID-19 and psoriasis, using three electronic medical databases–PubMed, EMBASE, Web of Science considering articles dated 1 January 2019 through 31 December 2021.

The search terms were selected to identify studies describing the relation between COVID-19 and psoriasis.

The keywords used were “COVID-19 AND psoriasis”, “COVID-19 AND psoriasis AND biologics”, “COVID-19 AND psoriasis AND systemic treatments”, “SARS-CoV-2 vaccinations AND psoriasis”.

All selected databases were searched from their respective inception, without any time interval. In addition, we searched by hand the reference lists of other relevant articles on COVID-19 and psoriasis.

In this first phase 298 records were identified from selected database. Records after duplicates removed were 237. Among the selected record, none was marked as ineligible by automation tools. Relevant studies were then selected. This process occurred in three phases. In the first phase, four researchers (A.C., F.D., E.M., G.R.) independently selected articles based on title. Any disagreements were resolved by consulting a senior researcher (A.O.). In the second phase, abstracts were evaluated. Two members of the research team (A.C., F.D.), independently evaluated each abstract. The research group resolved all discrepancies through unanimous consent. Ninety-five articles were excluded and 142 were evaluated for full-text analysis. Among them 15 manuscripts were not retrieved.

The third phase consisted of critical appraisal of the full text of the 127 selected papers. Ultimately, 82 studies were selected for the final qualitative synthesis. To be included into our review, studies had to be focused on COVID-19 course in psoriasis patients, on psoriasis course in patients affected by COVID-19, on effect of vaccines against SARS-CoV-2 infection in psoriasis patients, on systemic treatment strategies in psoriasis patients during pandemic. All included studies had to be published in English, with abstract available. No restrictions on study design were considered, and randomized controlled trial, case-control study, cross-sectional study, and case series were included. Articles were excluded from our review for only three reasons: reason 1 = review article (12 reports had been excluded following this reason), reason 2 = case reports (20 reports had been removed for this reason), reason 3 = reports in languages other than English (13 reports had been neglected for this reason). 

### 2.3. Data Extraction

A data extraction module was designed by A.C before data extraction to accelerate the entire process. To answer the research question, the following information was extracted from the included articles: Author(s) name and publication date; study design; study population; sample size; measured outcomes; study results; and study recommendations.

## 3. Results

**Figure 1:**
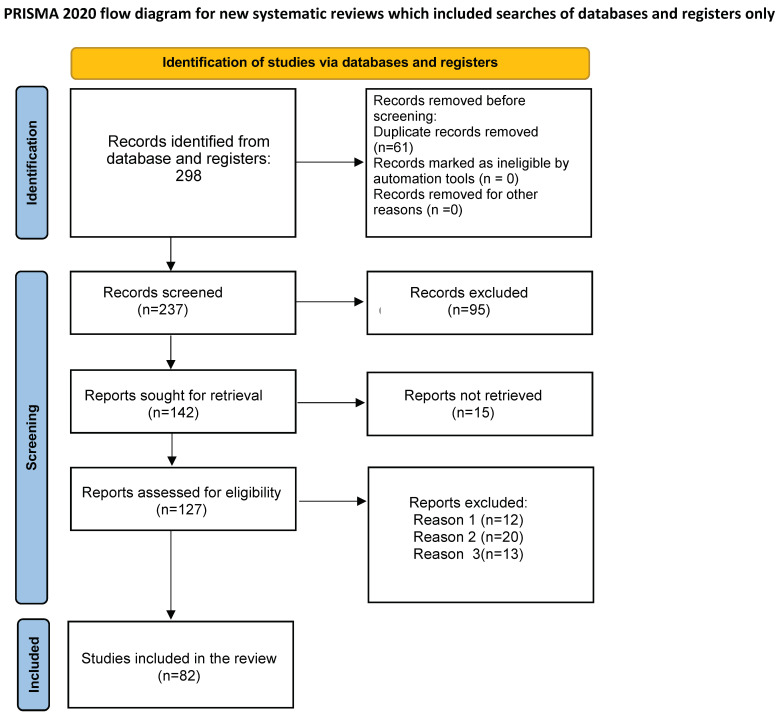
Preferred Reporting Items for Systematic Reviews and Meta-Analysis (PRISMA) on COVID-19 and psoriasis. From: Page MJ, McKenzie JE, Bossuyt PM, Boutron I, Hoffmann TC, Mulrow CD, et al. The PRISMA 2020 statement: an updated guideline for reporting systematic reviews. *BMJ* 2021, 372, n71. doi: 10.1136/bmj.n71 For more information, visit: http://www.prisma-statement.org/.

The flowchart of the PRISMA study is shown in Figure 1. Our search identified 140 records after removing duplicates. After review of titles and abstracts, 58 citations were dropped, and 82 were evaluated for full-text eligibility. After review of the full text, 40 case-control studies, case series studies, randomized controlled trials, and meta-analysis studies were found to be eligible and included in this study. 

Data found have shown that COVID-19 pandemic plays a conditional role in many clinical and therapeutic aspects of psoriasis patients management. Moreover, the increased clinicians experience during pandemic led to gradual changes in the paradigm of patient care.

### 3.1. Impact of SARS-CoV-2 Infection on Psoriasis Clinical Course

All selected studies are reported in Table 1.

Beyond the evidence that COVID-19 can present with cutaneous involvement, a growing concern about the potential exacerbation of preexisting psoriasis following COVID-19 emerged since the beginning of the pandemic. 

Viral infections are known to trigger the induction and exacerbation of psoriasis, and several mechanisms have been proposed to explain this process: antigen mimicry, epitope spreading, cytokine imbalance, and amplification of tissue destruction that increases the availability of self-antigens. In addition, SARS-CoV-2 infection is characterized by an overreaction of the immune response that could potentially shift the delicate immune balance in psoriasis [4].

A recent systematic review evaluated presentations, post-infection changes in the manifestation, diagnosis, and management of flare-ups of underlying dermatologic disease in patients with COVID-19. Authors reported nine patients with psoriasis and COVID-19, the flare-ups in five cases were attributed to either hydroxychloroquine or systemic corticosteroids which were initially considered promising treatments for COVID-19, and are well-known causes of drug-induced psoriasis [4].

The exacerbation of psoriasis in two cases could have resulted from the discontinuation of treatments (secukinumab and cyclosporine). In one case the COVID-19 treatment was not mentioned, the exacerbation of psoriasis in only one case could be confidently linked to COVID-19.

In the literature, cases of development and exacerbation of pustular psoriasis (PP) during COVID-19 infection have also been reported [5,6,7,8]. Mathieu et al. described a case of a 62-year-old woman with a positive family history of psoriasis who developed a new PP 2 weeks after resolution of symptoms of SARS-CoV-2 infection [5]. An additional case of a patient who developed a pustular psoriasis flare following SARS-CoV-2 infection was described in November 2020. To be fair, the patient had been taking hydroxychloroquine before the flare, but given that he had taken the drug in the past without any consequences for psoriasis, the authors attributed the PP flare to the viral infection [6]. Then, Dadras et al. described a 60-year-old male patient with a childhood history of psoriasis who developed generalized PP (GPP) 26 days after the onset of initial COVID-19 symptoms [7]. Finally, Samotij et al. reported the case of a 62-year-old patient with a history of Acrodermatitis of Hallopeau who developed pustular psoriasis after SARS-CoV-2 infection [8,9].

Thus, of all the cases in the literature, only a few can be assumed to have psoriasis induced by SARS-CoV-2 infection. This is not surprising, as viral infections have always been considered possible triggers of psoriasis onset [1]. Some authors hypothesize that psoriasis inflammation is linked to the virus’ production of inflammation-related cytokines, including IL-2, -7, and -10, granulocyte-colony stimulating factor, interferon-inducible protein 10, monocyte chemoattractant protein 1, macrophage inflammatory protein 1 alpha, and tumor necrosis factor α (TNFα) [10], all of which are known molecules in the development of psoriatic disease.

In summary, although there are not enough scientific evidence to demonstrate this phenomenon and no studies have been conducted to assess its prevalence, it cannot be ruled out that COVID-19 may exacerbate existing psoriasis, trigger psoriasis de novo, or change the phenotype of the disease (Table 1). 

### 3.2. Impact of Psoriasis on SARS-CoV-2 Infection 

All studies selected for this topic are summarized in Table 2. 

Skin has been described as one of the most common involved target organ in SARS-CoV-2 infection [11]; however, whether and how psoriasis is influenced by SARS-CoV-2 infection is still unclear, as published studies are rare and conflicting.

First, Dadras et al. hypothesized a strict connection between psoriasis and COVID-19 disease focusing their attention on the role of angiotensin-converting enzyme (ACE). 

In humans, ACE2 receptor is widely expressed in the lung, heart, kidney, endothelium and intestine, and also in the basal epidermal layer and sebaceous gland cells in normal skin [22].

It has already been demonstrated that SARS-CoV spike protein has a strong binding affinity to the human ACE2 receptor, and this could explain why skin may be a possible specific target for the SARS-CoV-2, which in fact can be also isolated from skin specimens [11,23,24,25,26].

Moreover, serum level of ACE is increased among psoriasis patients, and directly correlates with increased risk of cardiovascular comorbidities, including subclinical atherosclerosis [27]. 

In patients with severe psoriasis (e.g., erythrodermic) the increased ACE skin activity decreases after UVB treatment [28]. 

Conversely, the ACE inhibitors administration in patients with personal history of psoriasis may be associated with psoriasis exacerbation [22].

The link between ACE2 receptor and SARS-CoV-2 could result in ACE2 downregulation and in over-production of angiotensin by the ACE enzyme, the opposing physiological homolog of ACE2 [12]. 

Overall, the overexpression of ACE in COVID-19 patients may aggravate psoriasis favoring also cardiovascular complications, particularly in patients with severe psoriasis [13]. 

According to Dadras et al., psoriasis patients may be at a higher risk of worsening cardiovascular events in case of COVID-19 infection [22,29]. These findings, however, failed to be confirmed by subsequent studies. 

Patrick et al. [30] published an epidemiological analysis of 435,019 patients managed in Michigan Medicine (formerly the University of Michigan Health System) who had at least one health system encounter between 1 January 2019 and 20 June 2020. Authors made a genome-wide association study on transdisease meta-analysis between COVID-19 susceptibility and two skin diseases (psoriasis and atopic dermatitis).

People affected by an inflammatory skin disease have increased risk of being infected with SARS-CoV-2. 

Differently from results of Dadras et al. [22], having an inflammatory skin disease decreased the risk of requiring mechanical ventilation [30]. 

Authors postulated on the existence of different immunological rates of viral response in patients with inflammatory skin diseases, owing to the continuing activation of immunologic system [31,32,33,34,35].

Finally, more recent studies, focusing on patients with psoriasis receiving oral or biologic treatment conclude that patients with psoriasis show similar rates of infection with SARS-CoV-2 and COVID-19 prognosis as the general population, supporting our initial recommendation [14,15,36].

The same has been observed for psoriatic arthritis (PsA) [16,17,18]. 

Severity of COVID-19 is primarily driven by smoking status, sex (male), older age, and underlying comorbidities [19,20,21,37,38].

Moreover, National Psoriasis Foundation guidelines on the management of psoriatic disease during the pandemic (Version 2), confirm that age, male sex, and pre-existing comorbidities are crucial drivers for poor COVID-19 outcome in patients with psoriasis [38,39].

### 3.3. Impact of SARS-CoV-2 Infection on Systemic Treatments in Psoriatic Patients

All retrieved data are reported in Table 3. 

SARS-CoV-2 infection can be associated with increased, aberrant, and ineffective host immune response causing the acute lung injury (ALI) and acute respiratory distress syndrome (ARDS) typical of the serious forms of COVID-19 [58,59]. 

Five hyperinflammatory cell subtypes leading the inflammatory storm in lung injury, have been identified by Ren et al. [60]: a subtype of macrophage (Macro_c2-CCL3L1), three subtypes of monocytes (Mono_c1- CD14-CCL3, Mono_c2-CD14-HLA-DPB1, and Mono_c3-CD14-VCAN), and neutrophils. 

These hyper-inflammatory subtypes produce specific cytokines, Macro_c2-CCL3L1, specifically expresses CCL8, CXCL10/11, and interleukin (IL)-6; Mono_c1-CD14-CCL3 expresses high levels of IL-1β, CCL20, CXCL2, CXCL3, CCL3, CCL4, HBEGF, and tumor necrosis factor (TNF); and neutrophils express cytokines including TNFSF13B, CXCL8, FTH1, and CXCL16 [61].

Auto-inflammation driven by T cells, TNF-α, IL-23, and IL-17 underlies the pathogenic pathway of psoriasis [62]. Conventional systemic immunosuppressant, small molecules, and biological agents are commonly used for therapeutic management of moderate to severe psoriasis [63]. 

Several studies have already shown that psoriatic patients treated with systemic or biological immunosuppressants are exposed to a higher overall infection rate [40,48,59,60,61,62,63,64,65].

However, these studies did not consider the risk of virus infection separately [41] and none of them has considered the specific risk for coronaviridae family. Moreover, some data from literature have shown the safety of biologics in patients suffering from chronic viral infection as HIV [66]. 

Moreover, recently published data suggest that SARS-CoV1 may be associated with specific and distinct immune activation, unlike other respiratory viruses [67]. 

For all these reasons it is not appropriate to remark the susceptibility to SARS-CoV-2 in psoriatic patients based on these previous studies, but it is mandatory to understand the specific risk in psoriatic subjects under systemic therapies. 

Our review summarizes information currently available about the impact of systemic treatments on risk of SARS-CoV-2 infection and severe COVID-19 outcomes in psoriatic patients, according to different systemic drugs. 

Piaserico et al. [68] in a cohort study demonstrated that COVID-19 infection, hospitalization, and mortality rates were not increased in psoriatic patients on biologic treatment compared to the general population as reported in the following results: COVID-19 incidence rate (IR) was 9.7 (95% CI 3.9–20.1) per 10,000 person-months in a cohort of 1830 patients vs. 11.5 (95% CI 11.4–11.7) per 10,000 person-months in the general regional population. The IR of hospitalization for COVID-19-related pneumonia and COVID-19-related death in the same cohort of psoriatic patients compared with the general population was 6.5 (95% CI 2.0–15.6) and 0 (95% CI 0–10.4) per 10,000 person-months vs. 9.6 (95% CI 9.4–9.7) and 1.16 (95% CI 1.10–1.21) per 10,000 person-months, respectively. Mahil et al. [37] showed that the risk of COVID-19-related hospitalization is lower in patients treated with biologic drugs than in those treated with nonbiologic systemic therapies. A recent multicenter study collecting demographic data and disease characteristics of 1322 patients with psoriasis has been published; however, the results of the study did not demonstrate a statistically significant difference in COVID-19-related hospitalization between psoriatic patients using biologics (*n* = 9) and those not using biologics (*n* = 14) [42].

#### 3.3.1. TNF Alpha Inhibitors

TNF alpha inhibitors are the cornerstone in the treatment of psoriasis with biologics [69]. 

As increased serum levels of TNF alpha have been detected in many patients with severe COVID-19 compared to subjects with mild disease, the use of TNF-α inhibitors was proposed at the beginning of the outbreak with therapeutic purpose against COVID-19 [43,70]. 

Recently, Kridin et al. conducted a population-based cohort study in Israel to evaluate the risk of COVID-19 infection, COVID-19-associated hospitalization, and mortality among patients with psoriasis treated by TNF alpha inhibitors compared with psoriatic patients receiving other systemic agents; in detail, psoriatic patients under treatment with TNF alpha inhibitors (*n* = 1943), with those treated by methotrexate (*n* = 1929), ustekinumab (*n* = 348), and acitretin (*n* = 1892) concerning all previously mentioned COVID-19 outcomes. The risk of COVID-19 infection was comparable among subjects treated with TNF alpha inhibitors vs. methotrexate, ustekinumab, and acitretin. Exposure to TNF alpha inhibitors reduced the risk of COVID-19-associated hospitalization compared to methotrexate and ustekinumab, but not with acitretin. Although TNF alpha inhibitors were associated with a decreased risk of COVID-19-related hospitalizations, no significant difference in COVID-19-associated mortality was observed among the treatment groups. These results support the maintenance of TNF alpha inhibitors treatment throughout the pandemic. Patients with moderate to severe psoriasis who require systemic treatment during the pandemic can safely resort to TNF alpha inhibitors [45]. 

#### 3.3.2. IL17A/IL17R Inhibitors

As with TNF alpha, mean serum levels of IL-17 in patients with COVID-19 have also been shown to be substantially higher than values detected in the general population. Based on this finding, it was thought that IL-17 might play an active role in the cytokine storm and impact the severity of COVID-19. Therefore IL-17 inhibitors have been proposed as promising drugs for the prevention of abnormal inflammation and acute respiratory distress in COVID-19 [44,46]. A clinical trial focusing on safety and efficacy of ixekizumab treatment for patients with COVID-19 is currently ongoing in China [47]. From the available data, there does not appear to be evidence that IL-17 inhibitors increase the risk of SARS-CoV-2 infection or result in more severe COVID-19. 

In the 136-week real-life study by Galluzzo et al. [71], 119 out of 151 patients with moderate to severe plaque psoriasis continued treatment with secukinumab during the pandemic and none developed confirmed SARS-CoV-2 infection. Several case reports are described in the literature. Balestri et al. [49] reported one psoriasis patient infected with SARS-CoV-2 completely asymptomatic during ixekizumab induction phase who recovered from COVID-19 without any specific treatment one month later. Mugheddu et al. described two psoriatic patients infected with SARS-CoV-2 under long-term secukinumab. Both patients rapidly recovered from the infection between the two scheduled doses of secukinumab [50]. Di Lernia et al. described the case of an elderly psoriatic patient, affected by hypertension, who contracted mild COVID-19 during treatment with secukinumab with a favorable outcome. Notably, anti-IL17 antibody therapy was administered precisely during or shortly after the contagion [51]. 

#### 3.3.3. IL-23 Inhibitors

Differently from TNF-α and IL-17, IL-23 does not seem to have a major impact on anti-viral immunity, thus it seems not able to complicate SARS-CoV-2 infection [72]. 

A multicenter study including 57 patients monitored during the first 4 months of the pandemic in Central Italy found no increase in the SARS-CoV-2 infection rate during therapy with IL-23 inhibitors. In this study, only one patient (1.8%) experienced upper respiratory tract infection; three patients (5.3%) had contact with SARS-CoV-2- infected subjects; and none among them developed SARS-CoV-2 disease [41]. Several case reports reinforce the evidence that exposure to IL-23 inhibitors does not carry the risk of a more severe disease course or with more serious outcomes. Patients who suffered from COVID-19 ongoing anti-IL-23 therapy achieved full recovery from COVID-19, remained asymptomatic or developed only mild symptoms, although some of them were at risk of severe COVID-19 development [73,74]. These results suggest that exposure to IL-23 inhibitors will not increase the SARS-CoV-2 infection rate or COVID-19 disease course in patients with moderate to severe psoriasis. 

#### 3.3.4. Cyclosporine

Cyclosporine (CsA) is capable of inhibiting several viruses, including two very life-threatening viruses such as severe acute respiratory syndrome virus (SARS-CoV) and Middle Eastern respiratory syndrome coronavirus (MERS-CoV) [52]. For this reason, a potential beneficial role of CsA during the COVID-19 epidemic has been postulated [53,75]. 

Di Lernia and colleagues assessed the risk of contracting the COVID-19 infection and its severity in a group of 114 adult patients with psoriasis treated with CsA. Neither COVID-related deaths nor hospitalizations for COVID-19-related interstitial pneumonia have been reported in this study. Two psoriatic patients reported mild respiratory symptoms, with no need for hospital admission. In these cases, a preventive suspension of CsA was proposed at the onset of symptoms until their complete remission and after the fever had disappeared for at least a week [54]. Although further data or larger studies need to be done to confirm the safety profile of CsA in psoriatic patients during pandemic, starting from this observation, there are not many evidence that promote preventive discontinuation of CsA during COVID-19 outbreak in patients with psoriasis who are already being treated.

#### 3.3.5. Methotrexate

Consistent data on the safety profile of methotrexate (MTX) in psoriasis during the pandemic are still lacking. However, data from the literature demonstrate that the overall frequency of contracting pneumonia in patients with psoriasis using methotrexate is 0.8% [55].

The use of methotrexate has in some rare cases been associated with more severe episodes of COVID-19. One case-control study on psoriasis reported an increased hospitalization risk independently associated with its use [76]. 

In another study including 104 psoriasis patients, no significant difference in COVID-19 severity between the 13 COVID-19 patients treated with methotrexate (10–22.5 mg/week) and the psoriasis patients who did not receive any systemic treatment was found [77]. A retrospective cohort analysis including 65 patients with psoriasis under treatment confirm no significant association of methotrexate use with SARS-CoV-2 infection rates, percentage of COVID-19-positive patients who required hospitalization, need for ventilator use, or mortality [56].

However, one study demonstrated that MTX use in psoriasis patients has been associated with an increased hospitalization risk from COVID-19, without increased mortality [76]. 

As the impact of MTX on COVID-19 infection is still not clear, despite MTX is one of the most used immunosuppressive agent for psoriasis, studies focusing on MTX on SARS-CoV-2 infection or COVID-19 evolution in patients with psoriasis under treatment are warranted and needed.

#### 3.3.6. Apremilast

Apremilast is a phosphodiesterase-4 inhibitor that has been authorized by the FDA for the treatment of psoriasis. It affects the function of innate and adaptive immune systems by increasing intracellular cyclic adenosine monophosphate levels [78]. At the moment, one clinical investigation evaluating Apremilast for treatment in COVID-19 patients is ongoing, and one documented case suggests that Apremilast may be useful in a patient exposed to SARS-CoV-2 [57]. Y. Lytvyn et al. published a retrospective analysis involving 402 previously identified psoriasis patients who were receiving Apremilast. There were no documented cases of COVID-19 [79]. These findings reveal that when compared to the general population, Apremilast usage for psoriasis did not increase the chance of getting SARS-CoV-2 infection [80]. Apremilast may reduce the possibility of a cytokine storm associated with SARS-CoV-2 infections by downregulating the production of pro-inflammatory cytokines known to be enhanced during SARS-CoV-2 infection, such as tumor necrosis factor-alpha (TNF-a), interleukin (IL)-17, and IL-23 [81].

A 45-year-old man with erythrodermic psoriasis who was using Apremilast 30 mg twice daily and prednisone 12.5 mg daily was recently reported to have gotten SARS-CoV-2 pneumonia. He continued to use Apremilast and recovered after six days of therapy with lopinavir/ritonavir 400/100 mg twice daily and intravenous ceftriaxone 2 g/day [82].

As a result, people on Apremilast may be less prone to acquire a dangerous type of COVID-19 infection.

### 3.4. Impact of SARS-CoV-2 Vaccines on Patients with Psoriasis

Retrieved studies are reported in Table 4. 

Following SARS-CoV-2 vaccine delivery, a variety of cutaneous adverse effects have been recorded, including early or delayed site injection responses, maculopapular rash, erythema multiforme, perniones, and urticaria [83]. Along with these findings, the literature has reported on the effect of vaccination on the course of psoriasis.

Sotiriou et al. described 14 episodes of aggravation of psoriasis following COVID-19 immunization. Nine of them had a minor untreated history during the past year. Five patients were receiving topical therapy (steroids, calcipotriol/betamethasone), and the disease was well controlled. Exacerbation of psoriasis occurred in all patients shortly after vaccination with no difference in timing (mean, 10.36 days +/− 7.71) or severity between the vaccinations employed (50% mRNA technology vaccines and 50% adenovirus vaccine). Following immunization, five cases of psoriasis flare were treated with topical calcipotriol/betamethasone and nine cases with systemic drugs or phototherapy [84]. Psoriasis aggravation following SARS-CoV-2 vaccination can be related to the activation of humoral and cell-mediated immune responses, with Th17 playing a critical role [85,86].

Following that, Megna et al. documented 11 occurrences of psoriasis aggravation (8 male 72.7%, mean age 54.5 ± 8.9 years) following COVID-19 immunization with Pfizer mRNABNT162b2, Moderna mRNA-1273, or AstraZeneca-Oxford AZD1222 from February to July 2021, in contrast to Sotiriou. Contrary to Sotiriou et al., 54.5% of psoriasis flares associated with the COVID-19 vaccination occurred in individuals receiving biologic therapy. To control flares in these patients, topical calcipotriol/betamethasone and/or phototherapy were added to existing biologic therapy in four cases, while switching biologic agents was essential in the other two cases [87].

Musumeci et al. conducted a survey to determine the safety of SARS-CoV-2 vaccinations in patients with psoriasis who were receiving biologic therapy. Over a three-month period, 150 patients with stable plaque psoriasis who had been treated with biologics for at least two months were examined. Fifty patients (22 females and 28 males; ages ranged from 33 to 83 years) received just the first and second doses of SARS-CoV-2 vaccination. All patients were instructed to abstain from biological agents for ten days prior to and ten days following each dose of vaccination. A total of 24 patients received anti-TNF therapy, 14 got anti-IL17, 7 underwent anti-IL12-23 treatment, and 5 received anti-IL23. Following vaccination, all patients were examined for local and/or systemic side effects and/or potential adverse drug responses to SARS-CoV-2 vaccinations on days 2, 7, and 14. There were no adverse effects or psoriatic flares in any of the individuals. Only one patient treated with an infliximab biosimilar was referred to for psoriasis aggravation following vaccination. The remaining 100 patients stated they had not received the immunization as of yet [88].

Bostan et al. described two episodes of aggravation of plaque psoriasis following administration of COVID-19 vaccines inactivated and BNT162b2 mRNA. One instance occurred one month after the second dose of vaccination was administered (CoronaVac, China), and another happened after the first dosage was administered (Pfizer/Biontech, Germany), with extensive plaque expansion two weeks following the second dose of vaccine. Both individuals did not get systemic psoriasis therapy [89].

Onsun et al. described the beginning of generalized pustular psoriasis four days after receiving the first dose of the inactivated SARS-CoV-2 vaccine (CoronaVac, China) in a 72-year-old man who had previously been treated with topicals [90].

Lehhman et al. described a case of new-onset guttate psoriasis in a 79-year-old female following the BNT162b2 mRNA vaccination. The rash began 10 days after the first dosage and flared up following the second treatment. Topical calcipotriol/betamethasone ointment and UVB phototherapy have been used to treat flares [91].

Krajewski et al. described an aggravation of plaque psoriasis in a patient who had previously obtained full remission following deucravacitinib treatment during a clinical study. Five days after receiving the second dose of BNT162b2 mRNA SARS-CoV-2 vaccination, the patient developed psoriasis [92].

However, Kadali et al. observed that, in a group of 131 individuals with psoriatic arthritis, the PASI value remained constant in the majority of cases following immunization [93].

### 3.5. Vaccinal Hesitation and Strategies to Fight It in Psoriatic Patients

The international scientific community’s worry regarding vaccine hesitancy (apprehension about receiving a vaccination) and vaccine resistance (refusal to get a vaccine) is growing as new variants of SARS-CoV-2 continue to develop.

At the present, a significant task in avoiding this phenomenon is to strengthen the vaccination campaign, with the goal of covering the greatest number of people possible in the face of growing disinformation. Dermatologists who are directly involved in assisting patients with moderate to severe psoriasis with immunosuppressive or immunomodulatory medications should be regarded active participants in this chess game.

Patients with moderate to severe psoriasis, particularly those on biologics, should be regarded vulnerable and at a high risk of developing severe forms of COVID, and hence should adhere to the vaccine campaign completely. However, these patients’ perceptions of being at high risk vary, and this should be viewed as a significant potential hazard for individuals who underestimate the crucial importance of vaccination in preventing evocation of a serious form of COVID-19.

The levers for increasing vaccination compliance and tactics for avoiding vaccine resistance might be numerous and interconnected. To begin, dermatologists should raise awareness among patients with moderate to severe psoriasis that they are at an increased risk of developing a severe form of COVID-19, as compared to the general population matched for other well-known risk factors [94].

Dermatologists should discuss vaccination adverse events with their patients, reminding them that these effects are reversible and investigating ways to manage and reduce them. They should avoid using so-called “crisis language” while presenting the mechanism of action of vaccinations to minimize selfish or competitive behaviors and to prevent undermining people’s sense of social support and caring [95,96]. By contrast, a “gain-frame language” should be promoted to highlight communal gains and future advantages to increase self-consciousness and hence vaccine compliance [96].

Trust in presently available vaccinations should be created and strengthened by open communication that focuses on key points such as “what is known”, “what is unknown”, and most importantly, “what efforts are being made to learn more”. Generally, the pandemic has endangered temporal, geographical, and normative security. Additionally, SARS-CoV-2 vaccines have been approved by the European Medicines Agency for emergency use in Europe and are currently undergoing post-marketing surveillance. In this setting, as data on the effectiveness and safety of vaccinations continue to accumulate and are swiftly recorded and shared, vaccination strategies must be adjusted to specific circumstances and new issues. To ensure that these issues are easily accessible to patients, strategic involvement with patient associations may be necessary.

National psoriasis patient associations could alleviate negative pressure on both hesitant and opposed patients by demonstrating that their voices are heard and that their perspectives are considered not only by clinicians, but also by other decision-makers involved in vaccination campaigns, thereby increasing trust in vaccination [97,98].

Additional steps to be taken include anticipating and managing disinformation. The WHO acknowledged the presence of a parallel “infodemic” to the pandemic. The term “infodemic” refers to the abundance of information available, which can make it difficult to determine whether sources are trustworthy [98].

Pre- and post-debunking strategies (i.e., educating people about the dangers of disinformation before it spreads and correcting misinformation after it occurs) are critical, as misinformation concerning this subject is increasing [99].

### 3.6. Teledermatology as Emerging Assistence Paradigm in Moderate to Severe Patients

The outbreak of the SARS-CoV-2 pandemic has promoted the use of telemedicine, so dermatologists have been encouraged to use it in an outpatient setting.

Among dermatological patients, those with chronic, plaque type, and stable disease on systemic/topical maintenance therapy may be considered as ideal candidates for telemedicine, and several authors have demonstrated both the effectiveness and feasibility of this care approach [100,101,102].

Preference for telemedicine among psoriasis patients has been investigated by Gisondi et al. who investigated the preference for telemedicine over in-person visit among psoriasis patients receiving biologics and the reasons behind their preferences.

A positive attitude toward telemedicine was found in almost half of the patients affected by psoriasis in maintenance therapy with biological drugs [103].

Main reasons for preferring telemedicine were saving time, feasibility, and safety issues related to the COVID-19 pandemic.

The preference for telemedicine was found to correlate directly with the age of the patients, with disease benignity, and grade of severity [104].

The remaining half part of the patients preferred the in-person visit owing to the need of directly interacting with the clinicians without any “technological barriers”. However, providing patients with training related to “telecommunication” technologies might increase patients’ adherence to telemedicine.

The most limiting barriers associated with telemedicine have been identified as follows: recruitment of technically skilled staff; resistance to change; costs for technical equipment; implementation of simple procedures to obtain reimbursement of visits; attitude of patients to use technology daily depending on age, educational level, and personal predisposition; written and verbal communication impairment [105,106].

A strength point connected to the use of teledermatology for the assistance of psoriasis patients is the reduction of indirect costs related to health care and the opportunity of offering assistance to patients unable to reach the care centers for fragility, geographical reasons, difficulty in moving.

Considering all the opportunity and limitations, data from literature highlight teledermatology as an effective tool to counter limitations during COVID-19 pandemic, they also recommend the need to continuously examine our operative models to understand patient preferences, overcome practice-driven barriers, and ensure the sound allocation of limited health care resources [107].

## 4. Discussion

In the context of the current COVID-19 pandemic, physicians treating patients with psoriasis are tasked with challenging decisions when initiating immunosuppressive therapy or altering the existing regimens for their patients. Moreover, they must be ready to understand the needs of patients undergoing vaccination and be aware of the reciprocal effects of psoriasis and SARS-CoV-2 infection.

Relationships between COVID and psoriasis can be synthesized as follows:

- Most of the existing evidence is based on spontaneous case series, and although data from literature globally indicate no significant increase of the risk for severe COVID-19 evolution in psoriasis patients, it is hard to evaluate which biological agent is safer for COVID-19. However, from the current unclassified research, biologics do not seem to have a significant impact on the COVID-19 course. Regardless of which biological agents have been used, patients in biologics treatment seem to be not at higher risk for severe COVID complications.
- According to the recommendations of the major global dermatological associations [108,109,110], asymptomatic patients in close contact with a confirmed or probable COVID-19 case in the last 14 days can continue biologic therapy. It is advisable to discontinue or postpone biological treatment in symptomatic patients with confirmed SARS-CoV-2 infections until COVID-19 is completely resolved.
- For all candidates undergoing biological treatment, it is advisable to carefully assess the balance between benefits and risks of treatment for each patient.
- The National Psoriasis Foundation COVID-19 Task Force, in the last version of guidance for the management of psoriatic disease during the pandemic has reported that patients not infected with SARS-CoV-2 should continue biologics for psoriasis in most cases. Shared decision-making process between clinician and patient is recommended to guide discussions about the use of systemic therapies during the pandemic [58].

Scientific dermatological associations recommend that psoriasis patients with asymptomatic SARS-CoV-2 infection and at low risk for severe COVID-19 evolution (younger age, no systemic comorbidities) should continue their biological therapy. For high-risk patients (older age, with comorbidities, or metabolic disorders such as diabetes and obesity), the decision of discontinuing the treatment should be made case on case, through the balancing of benefits and risks, and the risk of disease relapse and retreatment failure should be taken into consideration too [41]. Similarly, Apremilast seems to be a safe treatment for psoriasis patients with COVID-19, thus at this moment it is not indicated to suspend the on-going treatment in psoriasis patients who get SARS-CoV-2 infection.

- Conversely, as the impact of MTX or CsA on COVID-19 infection is still to be cleared, despite they are commonly used immunosuppressive agent for psoriasis, studies focusing on these drugs on SARS-CoV-2 infection or COVID-19 evolution in psoriasis patients under treatment are few and inconclusive. Further research in this field is warranted and needed, before giving specific recommendation on continuation of their use in psoriatic patients affected by COVID-19.
- Although psoriatic patients have no increased risk of evolving toward severe forms of COVID-19 compared to the general population, they are frail subjects with respect to SARS-COV-2 infection which can favor the release of psoriasis clinically in remission, or favor its onset in predisposed patients or condition the appearance of different psoriasis phenotypes (e.g., pustular forms).
- Vaccination still represents the most effective measure to prevent the development of serious form of COVID-19 in psoriasis patients who should be advised to be vaccinated without discontinuing their biological treatment. In case the patient is starting a biological treatment, he should be vaccinated in advance, as current evidence suggests that the use of immunosuppressive agents may reduce the vaccine immune response to a certain extent [41].
- Dermatologists should be aware that vaccination of psoriasis patients is mandatory. They should consider the opportunity to be prompted to manage vaccination hesitancy in all patients, reassuring patients, with a clear and communicative language and informing them about the risks associated with vaccination, which are low in terms of the risk-benefit ratio [111].
- Although from the beginning of the pandemic, more and more information has been collected, and dermatologists globally improved their knowledge on managing psoriasis patients, the current evidence has still certain limitations. Therefore, it is necessary to be cautious when making clinical decisions. More prospective studies with higher levels of evidence are needed to support clinical decision-making.

## Figures and Tables

**Table 1:** Impact of SARS-CoV-2 infection on psoriasis clinical course.

Type of Study [Reference]	Outcomes	Number of Patients	Results
systematic review [3]	Evaluated presentations, post-infection change in the manifestation, diagnosis, and management of flare-ups	9 patients with psoriasis and COVID-19	5 cases of flare-ups due to hydroxychloroquine or systemic corticosteroids therapy for COVID-19 3 cases of exacerbation of psoriasis (2 resulted from the discontinuation of treatments 1 case due to COVID-19)
Case report [4]	New onset of pustular psoriasis (PP)	A case of a 62-year-old woman with family history of psoriasis	New PP 2 weeks after resolution of symptoms of SARS-CoV-2 infection
Case report [5]	New onset of pustular psoriasis (PP)	Patient with personal history of psoriasis	Administration of hydroxychloroquine before the flare
Case report [6]	New onset of generalized PP (GPP)	60-year-old male patient with personal history of psoriasis	New GPP 26 days after the onset of initial COVID-19 symptoms
Case report [7]	New onset of pustular psoriasis (PP)	62-year-old patient with a history of Acrodermatitis of Hallopeau	New PP after SARS-CoV-2 infection

**Table 2:** Impact of psoriasis on SARS-CoV-2 infection. NA: not applicable.

Type of Study [Reference]	Outcomes	Number of Patients	Results
Reviews, meta-analysis [11,12]	The role of angiotensin converting enzyme in the link between psoriasis and risk of the COVID-19	NA	Psoriasic patients may be at an higher risk of worsening cardiovascular events in case of COVID-19 infection
Epidemiological analysis [13]	Genome-wide association study transdisease meta-analysis between COVID-19 susceptibility and two skin diseases (psoriasis and atopic dermatitis).	435,019 patients	Having an inflammatory skin disease decreased the risk of requiring mechanical ventilation
Reviews, meta-analysis, National Psoriasis Foundation guidelines [14,15,16,17,18,19,20,21]	Prognostic factors of COVID-19 outcome in psoriatic patients	NA	Severity of COVID-19 is primarily driven by smoking status, sex (male), older age, and underlying comorbidities; Age, male sex, and pre-existing comorbidities are crucial drivers for poor COVID-19 outcome in patients with psoriasis

**Table 3:** Impact of SARS-CoV-2 infection on systemic treatments in psoriatic patients.

Type of Study [Reference]	Outcomes	Number of Patients	Results
Cohort study [40]	COVID-19 infection, hospitalization, and mortality rates in psoriatic patients on biologic treatment	1830 patients	COVID-19 infection, hospitalization, and mortality rates were not increased in psoriatic patients on biologic treatment compared to the general population. COVID-19 incidence rate IR: 9.7 (95% CI 3.9–20.1) vs. 11.5 (95% CI 11.4–11.7) per 10,000 person-months; Hospitalization IR: 6.5 (95% CI 2.0–15.6) vs. 9.6 (95% CI 9.4–9.7) per 10,000 person-months COVID-19-related death IR: 0 (95% CI 0–10.4) vs. 1.16 (95% CI 1.10–1.21) per 10,000 person-months
Global registry-based study [37]		374 clinician-reported patients	The risk of COVID-19-related hospitalization is lower in patients treated with biologic drugs than in those treated with nonbiologic systemic therapies
Multicenter study [41]		1322 patients with psoriasis	Not statistically significant difference in COVID-19-related hospitalization between psoriatic patients using biologics (*n* = 9) and those not using biologics (*n* = 14)
TNF alpha inhibitors			
Population-based cohort study [42]	Risk of COVID-19 infection, COVID-19-associated hospitalization, and mortality among patients with psoriasis treated by TNF alpha inhibitors compared with psoriatic patients receiving other systemic agents.	Psoriatic patients treated with: TNF alpha inhibitors (*n* = 1943) methotrexate (*n* = 1929) ustekinumab (*n* = 348) acitretin (*n* = 1892)	The risk of COVID-19 infection was comparable among subjects treated with TNF alpha inhibitors vs. methotrexate, ustekinumab and acitretin; Exposure to TNF alpha inhibitors reduced the risk of COVID-19-associated hospitalization compared with methotrexate and ustekinumab, but not with acitretin No significant difference in COVID-19-associated mortality was observed among the treatment groups
IL17A/IL17R inhibitors			
Clinical trial [43]	Safety and efficacy of ixekizumab treatment for psoriatic patients with COVID-19	recruiting	No evidence that IL-17 inhibitors increase the risk of SARS-CoV-2 infection or result in more severe COVID-19
Case report [44]		Psoriatic patient infected during ixekizumab induction phase	Recovered from a completely asymptomatic SARS-CoV-2 infection after 1 month without specific treatment
136-week real-life study [45]	Safety of secukinumab treatment for psoriatic patients with COVID-19	151 patients with moderate to severe plaque psoriasis	119 out of 151 patients continued treatment with secukinumab during the pandemic and none developed confirmed SARS-CoV-2 infection
Case series [46]		2 psoriatic patients infected with SARS-CoV-2 under long-term secukinumab	Recovered from the infection between the two scheduled doses of secukinumab
Case report [47]		Elderly psoriatic patient with hypertension	Recovered from mild COVID-19 during treatment with secukinumab with a favorable outcome
IL-23 inhibitors			
Multicentric study [48]	Safety of IL-23 inhibitors treatment for psoriatic patients with COVID-19	57 patients with moderate to severe plaque psoriasis monitored during the first 4 months of the pandemic.	In this study, only one patient (1.8%) experienced upper respiratory tract infection; three patients (5.3%) had contact with SARS-CoV-2- infected subjects, and none among them developed SARS-CoV-2 disease
Case report [49]		32-year-old woman under Guselkumab	Full recovery from COVID-19, remained asymptomatic
Case report [50]		40-year-old under Guselkumab	Full recovery from COVID-19 with development of mild symptoms
Case report [51]		45-year-old man under Risankizumab	Full recovery from COVID-19, remained asymptomatic
Cyclosporine			
Observational cohort study [52]	Safety of cyclosporine treatment for psoriatic patients with COVID-19	114 adult patients with psoriasis under cyclosporine	Neither COVID-related deaths nor hospitalizations for COVID-19-related interstitial pneumonia have been reported Two psoriatic patients reported mild respiratory symptoms, with no need for hospital admission: a preventive suspension of CsA was adopted at the onset of symptoms until their complete remission
Methotrexate			
Case–control study [53]	Safety of MTX treatment for psoriatic patients with COVID-19	3151 patients with psoriasis tested positive for COVID-19	Increased hospitalization risk inde-pendently associated with MTX
Observational cohort study [54]		104 psoriasic patients under MTX	No significant difference in COVID-19 severity between the 13 COVID-19 patients treated with methotrexate (10–22.5 mg/week) and psoriasis patients not receiving any systemic treatment
Retrospective cohort analysis [55]		65 psoriatic patients under MTX	No significant association of methotrexate use with SARS-CoV-2 infection rates, percentage of COVID-19-positive patients who required hospitalization, need for ventilator use, or mortality
Apremilast			
Retrospective analysis [56]	Safety of Apremilst treatment for psoriatic patients with COVID-19	402 psoriatic patients under apremilast	No documented cases of COVID-19
Case report [57]		45-year-old man with erythrodermic psoriasis	Affected by SARS-CoV-2 pneumonia. He continued Apremilast and recovered after six days of therapy with lopinavir/ritonavir 400/100 mg twice daily and intravenous ceftriaxone 2 g/day

**Table 4:** Impact of SARS-CoV-2 vaccines on patients with psoriasis.

Type of Study [Reference]	Outcomes	Number of Patients	Results
Case series [80]	Aggravation of psoriasis following COVID-19 immunization	14 episodes	Exacerbation of psoriasis occurred in all patients shortly after vaccination with no difference in timing (mean, 10.36 days +/− 7.71) or severity between the vaccinations employed (50% mRNA technology vaccines and 50% adenovirus vaccine) 9 cases treated with systemic drugs or phototherapy 5 patients received topical therapy
Case series [83]		11 occurrences of psoriasis aggravation	2 cases of switch in biological agents 54.5% of psoriasis flares associated with the COVID-19 vaccination occurred in individuals receiving biologic therapy; topical calcipotriol/betamethasone and/or phototherapy added to therapy;
Survey [84]	Safety of SARS-CoV-2 vaccinations in patients with psoriasis under biologic therapy	50 psoriatic patients under biologic treatment (24 under anti-TNF therapy, 14 under anti-IL17, 7 under anti-IL12-23 and 5 under anti-IL23) received 2 doses of SARS-CoV-2 vaccination	1 episode of aggravation of psoriasis under infliximab biosimilar, following vaccination
Case series [85]	Safety of SARS-CoV-2 vaccinations in patients with psoriasis undergoing topical treatment	2 psoriatic patients receiving CoronaVac, China and Pfizer/Biontech, Germany respectively	one month after the second dosage of CoronaVac, China and two weeks after first dosage of Pfizer/Biontech, Germany, onset of extensive plaque expansion topically treated
Case report [86]		72-year-old man previously treated with topicals	Beginning of generalized pustular psoriasis four days after receiving the first dose of the inactivated SARS-CoV-2 vaccine (CoronaVac, China)
Case report [87]	New-onset of psoriasis following COVID-19 immunization	79-year-old female	New-onset of guttate psoriasis 10 days after the first dosage and flared up following the second dose of BNT162b2 mRNA vaccination. Recovered after calcipotriol/betamethasone ointment and UVB phototherapy
Case report [88]	Safety of SARS-CoV-2 vaccinations in patients with psoriasis treated with Deucravacitinib	Psoriatic patient in full remission	Aggravation of plaque psoriasis five days after receiving the second dose of BNT162b2 mRNA SARS-CoV-2 vaccination,
Observational cohort study [89]	Safety of SARS-CoV-2 vaccinations in patients with psoriatic arthritis PsA	131 patients with PsA	PASI value remained constant in the majority of cases following immunization
